# Screen failure in phase I HIV clinical trials in Soweto, South Africa: an opportunity for care

**DOI:** 10.1186/1742-4690-9-S2-P130

**Published:** 2012-09-13

**Authors:** F Laher, M Mamba, K Otwombe, GE Gray

**Affiliations:** 1 Perinatal HIV Research Unit, Johannesburg, South Africa

## Background

Reasons for screen failures are evaluated for three phase 1 HIV vaccine clinical trials recruiting healthy low-risk participants at the Perinatal HIV Research Unit: SAAVI102/HVTN073 and SAAVI103/HVTN086 (the first trials evaluating a Clade C vaccine, Novartis Subtype C gp140 with MF59 adjuvant boosting SAAVI DNA-C2 and SAAVI MVA-C vaccine) and IAVIB003/HVTN091 (Ad26 and Ad35 ENV vaccines). Recruitment strategies involved a pre-screening programme, clinics and community outreach.

## Methods

Protocol-specific eligibility was determined using assessments of understanding, risk behaviour, medical history, physical examination, blood and urine testing, and for HVTN073 and 086, electrocardiograms. Descriptive analysis and multivariate logistic regression of age, trial group and gender were performed.

## Results

Between June 2009-2012, 225 participants(females=24%), median age 22 years(IQR:20-25) were screened. Overall 53% were ineligible, 60% of females vs. 51% of males (p=0.2). Site screening-to-enrolment ratios for 073, 086 and 091 were 2.1:1, 2.3:1 and 1.7:1 respectively. Medical abnormalities contributed 59% (n=70) of ineligibility reasons, chiefly urine abnormalities (n=12/70 where eleven displayed microscopic blood/haemoglobin, seven with leucocyte esterase and one had proteinuria), abnormal ECG (n=12), raised liver enzymes (n=10), raised blood pressure (n=9), low white cells (n=8) and hepatitis B (HBsAg+ve) or C (anti HCV+) (n=7). Other criteria excluded 41%(n=49) e.g. incomplete screening before enrolment closure (n=16), high-risk sexual behaviors (n=15), inability to comply with protocol (n=11), enrolment in another study (n=3), substance abuse (n=2, both cannabis-users), and poor understanding (n=2). In multivariate analysis, increasing age (OR 1.081, CI:1.007-1.16, p=0.032) predicted ineligibility but gender did not (OR: 0.67, CI: 0.35-1.3, p=0.24). HVTN073&086 participants were more likely to be ineligible than HVTN091 (OR 2.2, CI:1.1-4.5, p=0.023).

## Conclusion

Screen failures in phase 1 vaccine trials in Soweto provide young people opportunities for care, especially through blood pressure, urine, risk behaviour and hepatitis B/C screening. Older participants and those in protocols stipulating ECG criteria were more likely to fail screening.

